# Effect of Oral Neuromuscular Training on Tracheostomy Decannulation and Swallowing Function in Patients with Severe Acquired Brain Injury: A Pilot Randomized Controlled Trial

**DOI:** 10.1007/s00455-025-10837-z

**Published:** 2025-05-19

**Authors:** Melanie Blichfeldt, Mohit Kothari, Jesper Fabricius

**Affiliations:** 1 Hammel Neurorehabilitation Centre and University Research Clinic, Department of Clinical Medicine, Aarhus University, Voldbyvej 15, 8450 Hammel, Denmark; 2 Department of Audiology and Speech Language Pathology, Kasturba Medical College Mangalore, Manipal Academy of Higher Education, Manipal, Karnataka India

**Keywords:** Tracheostomy, Decannulation, Swallowing disorder, Oral neuromuscular training, Neurorehabilitation, Acquired brain injury

## Abstract

There is a lack of evidence on training modalities for improving swallowing function in tracheostomized patients. The objective was to investigate the effect of oral neuromuscular training on decannulation and swallowing function in tracheostomized patients with acquired brain injury. A pilot randomized controlled trial with 22 patients, 11 in the intervention group and 11 in the usual care group. Inclusion criteria were: ≥ 18 years, cuffed tracheostomy tube at admission for rehabilitation, and Fiberoptic Endoscopic Dysphagia Severity Scale (FEDSS) ≥ 4 at admission. Primary outcome was days from baseline until decannulation. Secondary outcomes were swallowing function assessed with FEDSS, Penetration Aspiration Scale (PAS), and the Yale pharyngeal residue scale (Yale scale) at baseline and following 4 weeks intervention. Participants in the two groups were comparable at baseline with regards to demographics and functional level. Difference in time until decannulation in the two groups was expressed with a hazard ratio of 1.40 (95%CI: 0.57; 3.43) in favour of the IQoro group. Swallowing function improved statistically significant in the usual care group on both PAS and Yale scale, whereas improvements in the IQoro group was only observed in FEDSS. Investigating between group differences, there was a statistically significant difference in pharyngeal residue assessed with the Yale Scale—pyriform sinus in favour of usual care (p = 0.018). Training with IQoro did not facilitate improvements in early decannulation or swallowing function compared to usual care. On the contrary, results showed less improvements in pharyngeal residue in the IQoro group compared with the usual care group.

## Introduction

Dysphagia is a frequent complication following acquired brain injury (ABI) [1, 2] and is associated with high morbidity, malnutrition, as well as an increased risk of pneumonia and mortality [1–4]. Following weaning from mechanical ventilation, patients with severe dysphagia may still require a cuffed tracheostomy tube to protect their airways from aspiration [5]. However, tracheostomy tubes are often of great inconvenience to the patients as it not only affects speech and swallowing but is also a potential contributor to silent aspiration [6], because the presence of a tracheostomy tube impairs normal laryngeal elevation during swallowing [7]. With a trend towards earlier transition from intensive care units to inpatient rehabilitation units, there are an increasing number of patients with severe ABI presenting with tracheostomy tubes at inpatient rehabilitation units [8]. Therefore, one of the first goals of rehabilitation is decannulation as soon as the patient is medically stable [7, 9, 10].

One of the mechanisms of action for decannulation is safe swallowing [11, 12]. According to Hägg and Morris [13], swallowing function may be improved by using a hand-held acrylic training device. The device which is named IQoro® is inserted between the lips and teeth and pulled forward against lip pressure [13]. It is argued by Hägg and Morris [13] that training with IQoro activates all the muscles in the swallowing process, including the outer longitudinal muscles that run along the sides of the esophagus and fasten under the diaphragm [13]. A recent study has demonstrated a positive effect of training with IQoro in improving swallowing rate and reducing clinical signs of aspiration among older people in intermediate care [14]. Focusing on individuals with stroke, observational studies on IQoro have shown functional improvements in the face and improvements in swallowing capacity [15–17]. However, more research on the effectiveness is urgently warranted, since IQoro has been implemented in clinical practice in several European countries without having strong scientific evidence to support its application. Therefore, the aim of this study was to investigate the effect of oral neuromuscular training with IQoro on decannulation and swallowing function in patients with tracheostomy tubes due to ABI.

## Methods

### Study Design

A randomized controlled pilot study. Study participants were randomly assigned to either the IQoro group (intervention) or usual care (control group). A block randomization procedure was made to ensure that an equal number of participants were randomized to the two groups. The online software Graphpad was applied for this. Only the principal investigator who had no patient contact in the trial, had access to the randomization list. Eligible participants provided informed consent, and surrogate consent was provided by family members for those patients who were not in a state to provide informed consent at admission. The trial was conducted in accordance with the ethical principles outlined in the Declaration of Helsinki [18]. The study was registered in ClinicalTrials.gov (ID: NCT05235282) and was approved by the Danish National Medical Research Ethics Committee on the 11 th of March 2022 [ID: 2201567].

### Participants and Recruitment

Tracheostomized patients with severe ABI were recruited from May 2022 until April 2024 from a semi-intensive rehabilitation ward. Dysphagia was confirmed by fiberoptic endoscopic evaluation of swallowing (FEES), which is performed systematically within five days of admission for all patients at the ward. Eligible participants should meet the following inclusion criteria: age ≥ 18 years, cuffed tracheostomy tube at admission for rehabilitation, and Fiberoptic Endoscopic Dysphagia Severity Scale (FEDSS) [19] ≥ 4 at admission. Exclusion criteria were: Admitted for 3 weeks evaluation, lack of ability to cooperate with mouth closure, and contraindications for using IQoro, including trigeminal neuralgia, paraesophageal hernia and achalasia cardiae.

### Power Calculation

No previous studies have examined the effect of oral neuromuscular training with IQoro on decannulation from tracheostomy tubes. Therefore, the sample size calculation was based on a study by Hägglund et al. [14] where swallowing rates have been studied before and after oral neuromuscular training with IQoro versus usual care, among elderly people in intermediate care. Here, a post-intervention swallowing rate of 6.22 (+ −2.16) was found in the intervention group and 3.64 (+ −2.72) in the control group. Based on this, the power calculation showed that a total of 22 participants (11 per group) were necessary to achieve a statistical power of 80%, with a two-sided significance level of 0.05.

### Usual Care

The usual care group received standard management for dysphagia in tracheostomized patients, which included Facial Oral Tract Therapy (F.O.T.T.®), tactile stimulation and facilitation of swallowing, above cuff vocalisation, therapeutic interventions related to meal situations and oral hygiene [10].

### IQoro Training Protocol

**Fig. 1:**
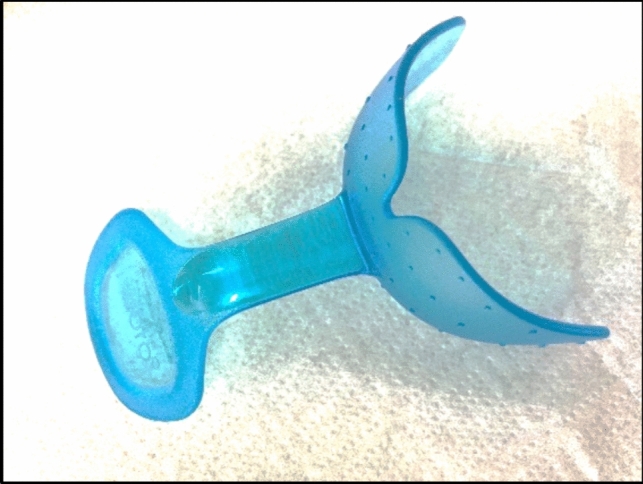
IQoro training device

The IQoro group, along with training with IQoro (MYoroface AB, Hudiksvall, Sweden), also received usual care. IQoro training was administered three sessions per day for 4 weeks. See Fig. 1. IQoro is inserted pre-dentally and pulled forward against lip pressure by the patient or an assistant [13]. Each session consisted of three repetitions, in which IQoro is held for 10 s between teeth and lips, followed by a short pause of 3 s. If the patient was unable to hold the IQoro, therapists were instructed to assist with the traction at the right angles in relation to the patient´s mouth. During the training, patients were instructed to sit on a chair or in bed with the body in an upright position with head support. The training was performed by trained occupational therapists. The date and time of each IQoro intervention was written in a weekly schedule, to assess treatment fidelity.

### Outcome Measures

The primary outcome was time from baseline assessment until decannulation from a cuffed tracheostomy tube based on the premise that safe swallowing is a strong prerequisite for decannulation [11, 12]. Patients were right censored at discharge from the ward or death. The weaning process from cuffed tracheostomy tubes follow a recently published action-oriented interdisciplinary weaning protocol developed at the ward. This protocol is based on FEES and several clinically documented criteria [10]. The weaning protocol is supported by use of a prognostic tool, which has also been developed at the ward [20]. Secondary outcomes were scales based on FEES assessment, which were scored at baseline and at the end of the 4 week intervention period, along with the Functional Oral Intake Scale (FOIS) and amount of secretion above the cuff. The scales applied for FEES were recently translated Danish Versions of the Penetration Aspiration Scale (PAS), FEDSS, and the Yale Pharyngeal Residue Scale (Yale Scale) [21]. These three scales are Likert scales which quantifies degree of swallowing function and safety, with lower scores representing better function. The PAS range 1–8, the FEDSS range 1–6, and the two Yale subscales range 1–5. The PAS was developed to provide reliable quantification of penetration and aspiration events observed during instrumental examination of swallowing function [22, 23], the FEDSS gives an indication of what type of nutrition and/or consistency can be recommended for the individual patient [19], and the Yale Scale quantifies the amount of residue in the pharynx (Vallecula residue or Pyriform sinus residue) [24].

FOIS is a Likert scale ranging 1–7, which reflects change in oral intake of food and liquid in patients with dysphagia, with higher scores representing better function. The FOIS has shown adequate reliability, validity, and sensitivity to changes in functional oral intake [25]. Daily secretion above the cuff was measured with a suction aid, which removes sub-glottic secretion [10]. Greater levels of daily amounts of saliva above the cuff is an indicator of aspiration risk [10].

### Data Analysis

Baseline characteristics were expressed as numbers (%), mean (± SD), or median (interquartile range), whichever was appropriate. Time until decannulation from a cuffed tracheostomy tube was illustrated in a Kaplan–Meier graph and analysed in a Cox proportional hazard model. In group- and between group differences on FEES scales were analysed using non-parametric signed-rank and ranksum tests, respectively. A two-tailed significance level of 0.05 was applied. Data visualisation and analyses were made with STATA v18/IC.

## Results

**Fig. 2:**
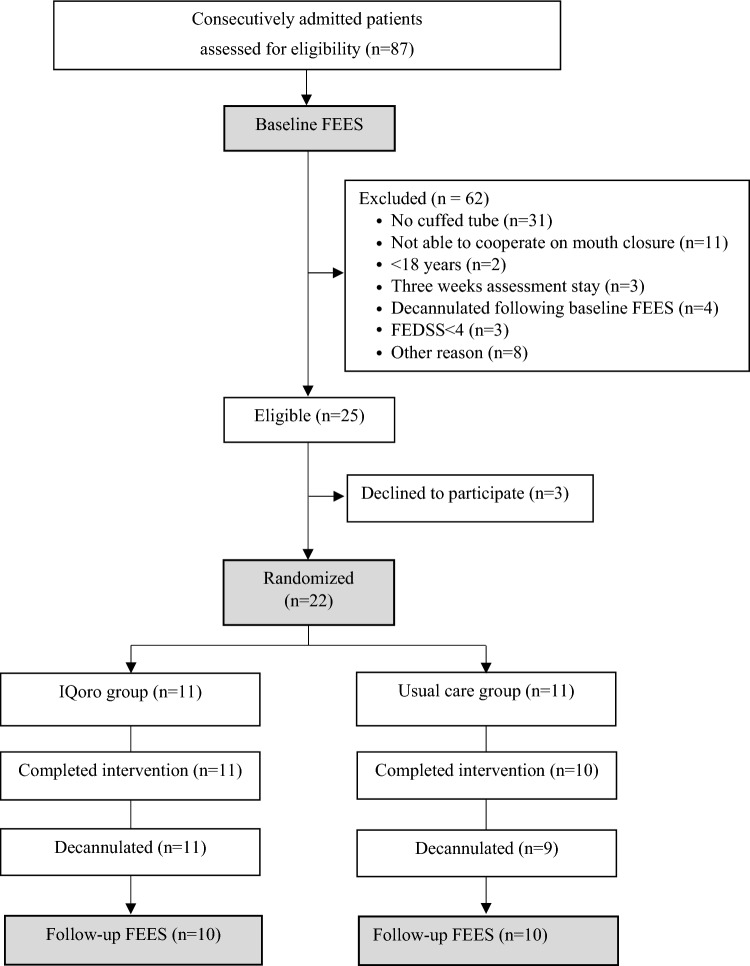
Study flowchart. Abbreviations: *FEES* Fiberoptic endoscopic evaluation of swallowing, *FEDSS* Fiberoptic Endoscopic Dysphagia Severity Scale

A total of 25 out of 87 consecutively admitted patients were eligible for the study, of which three declined to participate. Thus, 22 subjects were enrolled and 20 completed the entire study protocol (Fig. 2). As seen in Table 1, participants in the IQoro and usual care group were generally comparable at baseline, except that there was a borderline significant tendency showing that participants in the usual care group had more pharyngeal residue at baseline compared with the IQoro group, which is reflected in the Yale subscales. Participants in the IQoro group had a median of 82% adherence with IQoro training sessions. Lack of treatment fidelity was mostly attributed to participants sleeping, participants displaying agitated behaviour, and a couple of participants leaving the ward for a few days to have a percutaneous endoscopic gastronomy tube inserted.

**Table 1:** Baseline characteristics of participants in the IQoro-and usual care group

Characteristics	IQoro (n = 11)	Usual care (n = 11)	p-values
Age	63 (53–65)	57 (53–64)	0.491
Sex			1.000
Male	7	6	
Female	4	5	
Diagnosis			1.000
Ischemic stroke	2	2	
Hemorrhagic stroke	4	4	
SAH	4	4	
TBI	1	0	
Anoxic brain injury	0	1	
Injury until baseline, days	40 (± 11)	35 (± 12)	0.323
Scores at baseline			
FEDSS	5 (4–6)	6 (4–6)	0.775
PAS	6 (3–8)	8 (5–8)	0.259
Yale scale, vallecula	2 (2–3)	3 (2–4)	0.077
Yale scale, pyriform sinus	3 (2–3)	3 (2–5)	0.069
FIM	19 (19–26)	21 (18–22)	0.739
EFA	53 (37–53)	51 (49–53)	0.859
RLAS	4 (3–5)	5 (4–5)	0.386

Abbreviations: *SAH*   Subarrachnoid hemorrhage, *TBI*   Traumatic brain injury, *FEDSS*   Fiberoptic Endoscopic Dysphagia Severity Scale, *PAS*   Penetration Aspiration Scale, *Yale scale, vallecula*   Yale pharyngeal residue scale, vallecula residue, *Yale scale, Pyriform sinus*   Yale pharyngeal residue scale, Pyriform sinus residue, *FIM*   Functional Independence Measure, *EFA*   Early Functional Abilities, *RLAS*   Rancho Los Amigos Scale

**Fig. 3:**
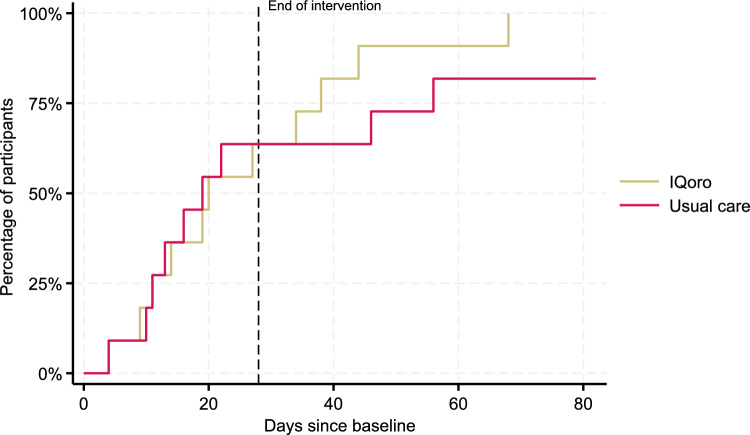
Time until decannulation in the IQoro and usual care group

In Fig. 3, the days from baseline until decannulation is displayed. The chances of being decannulated in the IQoro group compared to the usual care group is expressed with a hazard ratio of 1.40 (95%CI: 0.57;3.43) in favour of the IQoro group. Length of stay in the IQoro- and usual care group were 65 (± 32) days and 64 (± 24) days (*p* = 0.907), respectively. Two patients in the usual care group were right-censored in the Kaplan–Meier graph and the corresponding analysis; one patient who was still admitted with a cuffed tracheostomy tube, died after the end of the study protocol, and another patient who scored five on FEDSS and six on PAS at follow-up, was discharged with a cuffed tracheostomy tube.

**Fig. 4:**
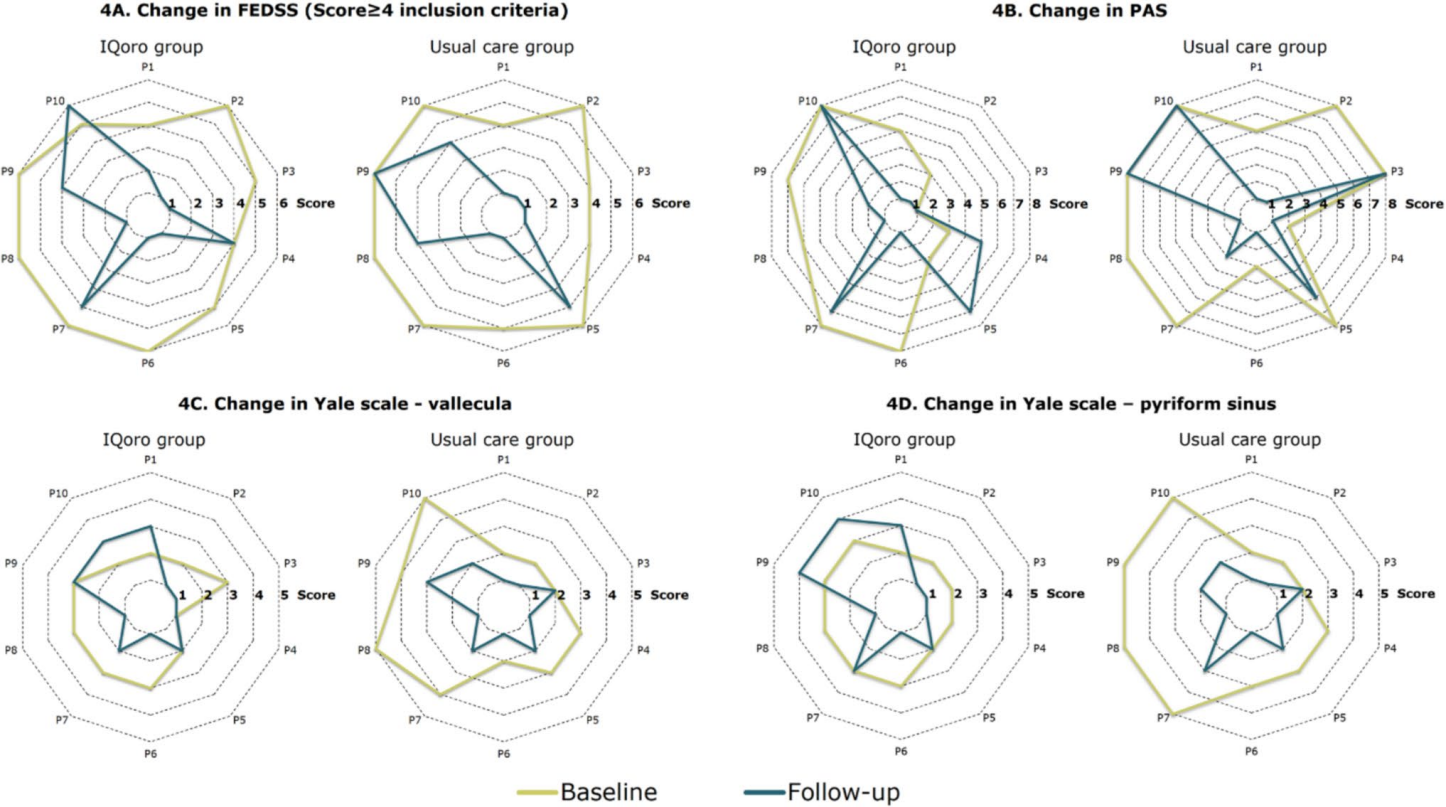
Change from baseline to follow-up in scales measuring swallowing ability and safety. Abbreviations: *P* Patient, *FEDSS* Fiberoptic Endoscopic Dysphagia Severity Scale, *PAS* Penetration Aspiration Scale, *Yale scale—vallecula* Yale pharyngeal residue scale, vallecula residue, *Yale scale–pyriform sinus* Yale pharyngeal residue scale, Pyriform sinus residue. Lower scores on all scales represent better function

A total of 20 participants, 10 in each group completed post-intervention FEES following the 4 weeks training protocol. One participant refused FEES at follow-up and one participant was decannulated and discharged before completing the 4 weeks training protocol. Swallowing function and swallowing safety assessed with FEES improved statistically significant in the usual care group on all scales, whereas improvements in the IQoro group was only seen on the FEDSS (Table 2). In analyses investigating between group differences, there was a statistically significant difference in pharyngeal residue assessed with the Yale Scale—pyriform sinus in favour of usual care. Raw data on scales used for quantifying swallowing function are presented in Fig. 4A–D. As seen, a few of participants in the IQoro group had worsened swallowing safety at follow-up.

**Table 2:** Swallowing function in the IQoro and usual care group at baseline and post intervention

	IQoro (n = 10)				Usual care (n = 10)				Between group difference
	Baseline	Post	Change	p-value	Baseline	Post	Change	p-value	p-value
FEDSS	6 (5–6)	2 (1–4)	3 (1–5)	0.012	6 (4–6)	1 (1–4)	3 (2–4)	0.006	1.000
PAS	6 (3–8)	2 (1–7)	2 (0–5)	0.123	8 (5–8)	2 (1–8)	2 (0–5)	0.011	0.540
Yale V	3 (2–3)	2 (1–3)	1 (0–2)	0.157	3 (2–4)	2 (1–2)	1 (1–2)	0.005	0.109
Yale P	3 (2–3)	2 (1–3)	1 (−1–1)	0.318	3 (2–5)	2 (1–2)	2 (1–3)	0.006	0.018

Abbreviations: *FEDSS*   Fiberoptic Endoscopic Dysphagia Severity Scale, *PAS*   Penetration Aspiration Scale, *Yale V*   Yale pharyngeal residue scale, vallecula residue, *Yale P.*   Yale pharyngeal residue scale, Pyriform sinus residue

All participants were nil by mouth at baseline, corresponding to FOIS 1. At discharge both the IQoro- and usual care group had improved oral intake with median FOIS 6 (IQR: 3–7), p = 0.006 and FOIS 6 (IQR: 6–7), p = 0.008, respectively. There was no difference in FOIS at discharge between groups, p = 0.523. Data on number of days with saliva above the cuff is of course dependent on how long patients had a cuffed tracheostomy tube. Thus, all participants, except one in the IQoro group, had daily measures of saliva above the cuff for at least one week following baseline. The mean millilitres of saliva above the cuff in the first week following baseline was 21 ml (± 10) in the IQoro group and 22 ml (± 17) in the usual care group, p = 0.913.

## Discussion

This study investigated the effect of oral neuromuscular training with IQoro on decannulation from a cuffed tracheostomy tube and swallowing function, in patients with severe ABI. Results showed that the IQoro group tended to have fewer days until decannulation compared to the usual care group, but the result were not statistically significant. Furthermore, through visual inspection of the Kaplan–Meier graph, it is also apparent that the differences between the two groups in time until decannulation were seen in the period following the 4 weeks intervention. Thus, making it more likely to have been caused by other factors than the intervention, such as differences in consciousness level, cough reflex and strength; along with active infections which are a contraindication of decannulation from a cuffed tracheostomy tube [20, 26].

Surprisingly, it was only the usual care group which improved on the PAS and Yale scale, which describes the risk of aspiration and amount of residue in the vallecula and pyriform sinus, respectively. It is noteworthy that the usual care group had less residue in pyriform sinus following the training protocol, compared with the IQoro group. This result points to a potentially harmful effect of IQoro training in tracheostomized patients. However, this might well be a chance finding due to the low number of participants, and findings needs to be replicated before conclusions may be drawn.

According to the International Classification of Functioning (ICF), swallowing is described as functions including both the oral, pharyngeal and oesophageal processes related to transporting food and drinks [27]. Some of the previous observational studies on IQoro by Hägg and Tibbling [15, 16] related to swallowing difficulties, have investigated the associations between training with IQoro and lip closure and facial functions [15, 16], but also swallowing capacity, which is a more collective process encompassing both the oral and pharyngeal processes of swallowing. More recently, Hägglund et al. [14] carried out a cluster-randomized study in which swallowing rate and aspiration risk was assessed. Decline in aspiration risk was seen in the IQoro group, but not in the control group. However, aspiration risk was based on unblinded subjective assessments based on a water swallowing test, and findings may therefore be biased [14]. In the present study, instrumental assessment of swallowing function was applied, which should provide more valid and objective assessments. Additionally, it should be considered that the intervention in the study by Hägglund et al. [14] was performed in 16 care units, and the usual care was performed in 18 other care units. Since usual care is not described in detail, results in favor of IQoro may also be due to differences in the usual care practice between care units. In the present study, the focus was on the pharyngeal phase of swallowing as the pivotal mechanism of action driving the weaning process from a cuffed tracheostomy tube [12], because it is argued by Hägg and Morris [13] that training with IQoro activates all the muscles in the swallowing process. However, the scientific rationale behind this assumption is ambiguous.

In relation to level of consciousness and cognitive functioning, participants in the study had median RLAS scores of four and five in the IQoro and usual care group at baseline, respectively. Thus, apart from having tracheostomy tube, they are a different patient group than those described in previous research on IQoro, which consist of community dwelling individuals with stroke and elderly individuals in care facilities [15–17]. Therefore, the results should be interpreted with caution, since level of consciousness have been associated with time to decannulation [20, 26].

Trained occupational therapists performed IQoro training for most participants in the present study providing more robustness to the training protocol, whereas training seemed to be mostly self-administered in the previous studies [15–17]. On the other hand, motivation to participate in the present study may be lower than previous studies, because most participants were not able to consent themselves due to fairly low level of consciousness and cognitive functioning at enrollment. In line with this, agitation was often the cause when IQoro training was not carried out. However, the study was carried out in a hospital setting in which a tight schedule was kept in order to complete the training protocol. Additionally, patients were only enrolled in the study if they could cooperate on mouth closure. Therefore, although motivation may have been less compared with previous studies, the adherence rate with the IQoro training protocol was deemed high on a group level [28].

In recent years there has been a large increase in available medical devices and it is anticipated that the global medical device market size will increase from 542 billion USD in 2024 to 886 billion USD in 2032 [29]. Based on this growth, it is challenging for research to keep up with advertisements for medical devices and consumer demands [30]. IQoro is a medical device which is boldly stated to be a treatment for the underlying cause of both swallowing difficulties, reflux, heartburn, snoring, and sleep apnoea [13, 31]. Based on this premise, IQoro is stated to have been used by more than 100,000 clients [31]. However, the scientific evidence for its effectiveness cannot keep up with the increasing demands. Thus, high-quality studies by research teams with no conflicts of interest is urgently needed for medical devices such as IQoro.

IQoro is not the only oral neuromuscular training device in the market. A cheaper alternative is Muppy®, which works in the similar way as IQoro, although it has been described that it does not produce the same vacuum in the mouth as IQoro [32]. The effect of Muppy on swallowing function have been investigated in a recent RCT including 40 individuals with first-time stroke [32]. A total of 20 participants received usual care for 5 weeks (orofacial-sensory-vibration stimulation), and 20 participants received usual care plus daily training with Muppy. There was no improvements on aspiration risk measured with PAS in neither the intervention or usual care group, and therefore also no between-group differences. Between-group differences in swallowing rate and lip force was seen at 12 months follow-up only, and these results are therefore likely to be due to other reason than the intervention. If IQoro and Muppy will show an effect on dysphagia in future studies, it would be relevant at to compare the effectiveness of IQoro and Muppy on dysphagia.

The present study has some strengths and limitations which deserves mentioning. Only 22 participants were enrolled, which was due to a power calculation based on the study by Hägglund et al. [14]. This calculation may be questioned due to potential bias in the study on which it was based, but the calculation was made due to lack of studies with populations and outcomes, which more closely resembled the present study. However, 22 participants fullfilling our inclusion criteria are difficult to come by, which is also illustrated in the flowchart, and also the reason why it took two years to enroll enough participants. It could be seen as a limitation that a very selective population of patients with tracheostomy tubes due to ABI were enrolled, because it limits generalisability of findings. However, it could also be seen as a strength since results show that IQoro may not be suitable for this particular patient category. IQoro was used sporadically for this patient category at the ward before the study, and the intervention is therefore a clear reflection of clinical practice at the ward. This also means that the results are a clear representation of the effect of IQoro in normal clinical practice, outside the frame of a clinical trial. Following the study, clinicians at the ward will now refrain from training swallowing function with IQoro for patients fulfilling the study eligibility criteria.

Decannulation and swallowing assessment was not done by assessors who were blinded to treatment allocation due to logistic reasons. However, FEES is a fairly objective assessment of swallowing function, on which decisions on the weaning proces is based, and blinded assessors are therefore not likely to have changed the results of the study. Additionally, it is obvious that it is not possible to blind participants, but since the patients had low consciousness and mostly did not consent to participate by themselves, it is not suspected that placebo and hawthorne effects have played any great role on the results.

### Clinical and Research Implications

In this study we found that four weeks of training with an oral neuromuscular training device named IQoro, as an add-on to usual care, was not superior to usual care alone, in improving swallowing function and reducing time until decannulation in patients with severe ABI. On the contrary, findings from the FEES showed a potentially harmful effect of IQoro with less improvements in pharyngeal residue in the IQoro group compared with the usual care group. However, this finding needs to be replicated before any conclusions may be drawn.

The study results should not be generalized to patient categories outside the eligibility criteria, since this is a highly selective population in which injury severity and consciousness level may also have an effect on swallowing function and successful decannulation. Future studies investigating the effect of IQoro and similar training devices on a more general population of non-tracheostomized patients with ABI are warranted, in order to have scientific evidence which can keep up with marketing strategies and consumer demands for products such as IQoro.

## Data Availability

Data is available upon reasonable request.
